# Association between COVID-19 Severity and Expression of Viral Nucleic Acid Sensor Genes in Peripheral Blood Mononuclear Cells and Nasopharyngeal Epithelial Cells

**DOI:** 10.4269/ajtmh.24-0004

**Published:** 2024-05-07

**Authors:** Zohreh-Al-Sadat Ghoreshi, Emad Behboudi, Hedyeh Askarpour, Fatemeh Raesi Nejad, Nasir Arefinia

**Affiliations:** ^1^ School of Medicine, Jiroft University of Medical Sciences, Jiroft, Iran;; ^2^ Department of Basic Medical Sciences, Khoy University of Medical Sciences, Khoy, Iran;; ^3^ Student Research Committee, Jiroft University of Medical Sciences, Jiroft, Iran;; ^4^ Bio Environmental Health Hazards Research Center, Jiroft University of Medical Sciences, Jiroft, Iran

## Abstract

This study examined expression of key viral nucleic acid sensor genes *MDA5, ZBP1,* and *AIM2* in nasopharyngeal epithelial cells and peripheral blood mononuclear cells (PBMCs) obtained from 153 COVID-19 patients across a spectrum of disease severity (mild, severe, and critical) and 42 healthy controls. Quantitative reverse transcription polymerase chain reaction was used to quantify and compare sensor transcript levels. The COVID-19 cohort had a mean age of 53.6 years. All three sensor genes including *MDA5* (3.2-fold), *ZBP1* (5.1-fold), and *AIM2* (4.7-fold) exhibited significantly higher messenger RNA expression in both nasopharyngeal and PBMC samples from infected patients compared with healthy controls. Furthermore, sensor transcript upregulation positively correlated with escalating disease severity. During early stages, ZBP1 and AIM2 transcripts were selectively elevated within the nasopharyngeal compartment, suggesting a localized antiviral response. Whereas later during critical disease stages, ZBP1 and AIM2 levels became preferentially heightened within circulating PBMCs, indicating systemic immune cell activation. By comparison, MDA5 elevation manifested within nasopharyngeal epithelial cells during both early- and late-phase infection. Intriguingly, males displayed higher ZBP1 and AIM2 expression compared with females, whereas MDA5 transcript levels were conversely higher among females. Overall, escalation of these key viral sensor genes appears closely linked to COVID-19 progression, with initial nasal mucosal upregulation transitioning to widespread blood cell activation in severe systemic disease. These patterns of sensor expression suggest frontline immunological efforts to constrain early viral invasion and combat severe late-stage COVID-19 illness through innate detection of replicating SARS-CoV-2.

## INTRODUCTION

COVID-19 has infected millions of people globally since its outbreak in late 2019, causing numerous deaths and unprecedented disruption to daily life.^1^ Despite the efforts of governments, healthcare providers, and scientists, it continues to pose a serious threat to global health and the economy.^2^^,^^3^

The recognition of viruses by host cells occurs through pattern recognition receptors (PRRs) such as nucleotide oligomerization domain–like receptors, Toll-like receptors, and retinoic acid-inducible gene–like receptors (RLRs). These receptors could activate signaling pathways that lead to the production of immune mediators, including interferons (IFNs) and pro-inflammatory cytokines, promoting immune defense against viral infections.^4^

Among the members of the RLR family, melanoma differentiation-associated protein 5 (MDA5) plays a critical role in detecting various RNA conformations, which is essential for triggering the production of IFN-I and IFN-III.^5^ Studies have shown that the presence of cytosolic MDA5 is vital for inducing IFN-I and IFN-III in lung epithelial cells after being infected with SARS-CoV-2.^6^^–^^8^ MDA5 is crucial in the recognition of picornaviruses, reoviruses, and coronaviruses, and recent studies suggest its potential involvement in the host response to COVID-19.^9^

Z-DNA binding protein 1 (ZBP1) has been implicated in the detection of viral RNA in infected cells and the initiation of antiviral responses.^10^ ZBP1 could trigger programmed cell death, which may involve pyroptosis, necroptosis or apoptosis, depending on caspase activity.^11^ There is evidence that ZBP1 is involved in the induction of inflammatory responses in COVID-19 patients.^12^ ZBP1 proteins have also been reported to induce programmed cell death in influenza A virus (IAV)-infected cells.^13^

Absent in melanoma 2 (AIM2), is a prominent protein responsible for regulating and modulating immune responses against various infections. Its vital function lies in orchestrating a robust immune defense against invading pathogens. AIM2 induces antiviral responses during pyroptosis by sensing host mitochondrial genomic DNA.^14^ In a previous study, it was shown that AIM2 transcript was significantly upregulated in lung tissue samples of COVID-19 patients compared with healthy control subjects.^15^ These findings suggest that AIM2 may play a role in the immune response to COVID-19.^16^

This study aimed to address two specific knowledge gaps: 1) How do expression patterns of critical viral sensor genes (*MDA5, ZBP1,* and *AIM2*) differ between immune cell populations (peripheral blood mononuclear cells [PBMCs]) and airway epithelial tissue in COVID-19 patients? Most prior work has focused solely on one cell type compartment when evaluating innate immune responses. Simultaneously examining both provides important spatial insights during infection. 2) Does expression of these sensor genes change across escalating severity during COVID-19 progression? Evaluating differential gene expression at early mild versus late critical phases can reveal pivotal temporal dynamics of antiviral immune response engagement. Although the virus mainly affects the respiratory system, recent data suggest that it also affects immune cells such as PBMCs. We hypothesized that the gene expression patterns of these PRRs may differ between these two compartments in COVID-19 patients and that this could provide important insights into the spatial and temporal dynamics of the host immune response to the virus. The results might help in developing more effective therapies for COVID-19.

## MATERIALS AND METHODS

### Patients.

Selection of patients and sampling in this case–control study was done in Afzali Hospital of Kerman University of Medical Sciences as the main center of control and treatment of COVID-19 patients in a period of ∼3 months (December 2022 to March 2023). Based on WHO classification,^17^ the COVID-19 patients were classified into three groups: critical, severe, and mild. In the present study, 153 patients were randomly selected among the patients who had the necessary criteria to enter in each group. Inclusion criteria were as follows: 1) COVID-19 patients with quantitative reverse transcription polymerase chain reaction (qRT-PCR)-confirmed SARS-CoV-2 infection; 2) throat swabs yielding positive results; 3) presenting with symptoms such as dehydration, vomiting or diarrhea, intense cough with purulent sputum or bloody, chest pain, and dyspnea; 4) not having received any COVID-19 vaccines; and 5) aged between 27 and 77 years. A control group of 42 healthy volunteers referred to health centers were matched for age and gender. The healthy control group did not have any diseases recorded in their health history, such as chronic viral or bacterial diseases, autoimmune diseases, or cancer. Written informed consent was also given by each participant. The local Ethics Committee of Jiroft University of Medical Sciences approved the study protocol (code IR.JMU.REC.1402.003).

### Criteria for COVID-19 severity.

The WHO classifies COVID-19 patients based on their symptoms and severity of illness as follows:

- Mild: patients with mild symptoms who do not have pneumonia or hypoxemia.
- Severe: patients with clinical signs of pneumonia (fever, cough, dyspnea) and saturation <90% on room air. May include shortness of breath, altered mental status, and so on.
- Critical: patients displaying respiratory failure, septic shock, and/or multiple organ dysfunction or failure. This can include respiratory failure requiring mechanical ventilation, shock, and combined failure of other organs.

The classification is based on clinical examination, symptoms, vital signs, and pulse oximetry readings to assess severity and categorize patients. This helps guide treatment decisions and care protocols. More severe classifications involve compromised respiratory/circulatory function and organ dysfunction, indicating critical care is needed. The WHO provides this case definition to standardize reporting and comparison of severity data globally.

### Sampling.

Nasopharyngeal swabs samples were collected from COVID-19-confirmed patients according to WHO standard protocol and using real-time RT-PCR targeting SARS-CoV-2–specific genes. Five milliliters of whole blood samples were collected from each participant to isolate PBMCs. PBMC isolation was performed using Ficoll (Lymphodex, Norway, Oslo) through density gradient centrifugation. Subsequently, they were aliquoted and stored at −70°C.

We extracted RNA from PBMCs and nasopharyngeal samples with Yekta Tajhiz (Yekta Tajhiz Azma Co., Tehran, Iran) and ROJE (ROJETechnologies, Tehran, Iran) commercial kits, respectively. Subsequently, the quantity and purity of RNA extraction were measured, and then complementary DNA (cDNA) was conducted with a cDNA synthesis kit (Yekta Tajhiz).

To measure the messenger RNA (mRNA) level of *MDA5*, ZBP1, and AIM2 genes, qRT-PCR was conducted with the Ampliqon Master Mix (Ampliqon, Denmark). Primer efficiencies across selected concentration ranges were calculated through standard curve analyses on known control templates. Only verified primer sets exhibiting appropriate efficiency and specificity profiles were used for quantitative gene expression experiments. The efficiencies of amplification were also determined and incorporated into data normalization by using β-actin transcript as an internal control. The qRT-PCR was conducted at specific temperatures with melting curve analysis to confirm its specificity (Table 1). The Pfaffl formula^18^ was used to compute the levels of target genes transcript.

**Table 1:** The MDA5, ZBP1, and AIM2 specific primer sequences, annealing temperature, and polymerase chain reaction (PCR) condition used

Gene	Sequence (5′-3′)	Annealing Temperature (°C)	PCR Protocol
*MDA5*	F: CAGTGGTTCAGGAGTTATCG	56.3	95°C, 5 min, 1×
			95°C, 1 min 57.4°C, 45 sec, 35× 72°C, 1 min
	R: GGTTATTCTTGTAATGCTTGGC		
			72°C, 5 min, 1×
*ZBP1*	F: CCAAGTCCTCTACCGAATGA	62.1	95°C, 5 min, 1×
			95°C, 1 min 62.1°C, 35 sec, 40× 72°C, 1 min
	R: TGTCTTCCTCCCGTTGTT		
			72°C, 5 min, 1×
*AIM2*	F: GCTGCACCAAAAGTCTCTCCTC	59.8	95°C, 5 min, 1×
			95°C, 1 min 59.8°C, 40 sec, 40× 72°C, 1 min
	R: CTGCTTGCCTTCTTGGGTCTCA		
			72°C, 5 min, 1×
Beta-actin (Endogenous control)	F: CACCATTGGCAATGAGCGGTTC	59.6	95°C, 5 min, 1×
			95°C, 1 min 60°C, 40 sec, 35× 72°C, 1 min
	R: AGGTCTTTGCGGATGTCCACGT		
			72°C, 5 min, 1×

min = minute; sec = second.

## STATISTICAL ANALYSES

Data statistical analysis and graph plotting were carried out by the SPSS 26 (SPSS, Chicago, IL) and GraphPad Prism 9 (GraphPad, San Diego, CA) software packages. Data were analyzed by one-way analysis of variance and Dunnett’s test and independent-sample *t*-test. *P*-values <0.05 were considered significant.

## RESULTS

From a total of 183 participants, 153 patients with COVID-19 (51 critical, 51 severe, and 51 mild) and 30 healthy controls were included in the study. There were 26 males and 25 females in each patient group. The control group had an equal distribution of 15 males and 15 females. The mean age of all patients and healthy control groups are listed in Table 2.

**Table 2:** The mean age of the patients and control group

Group	Age			*P*-Value
	Mean	SEM	IQR	
Control	48.65	2.7	24.0	0.4
Total cases	53.6	3.3	23.7	
Mild	54.75	1.9	21.65	0.9
Severe	52.23	1.56	23.5	
Critical	53.42	1.2	21.7	

IQR = interquartile range; SEM = standard error of the mean.

### The level of target mRNA gene in all COVID-19 patients and healthy group.

**Figure 1.**
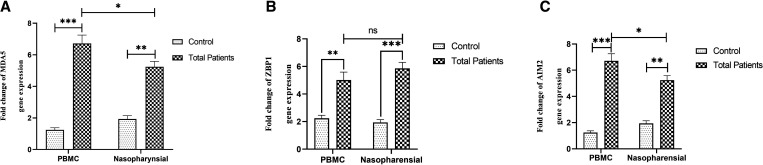
Comparison of MDA5 (**A**), ZBP1 (**B**), and AIM2 (**C**) transcript in the PBMCs and nasopharyngeal samples of all COVID-19 patients and the healthy group. The data is presented as fold change and differences in expression between the two groups are indicated by *P <0.05, **P <0.001, and ***P <0.0001. ns = not significant.

As shown in Figure 1, the fold-change expression of MDA5 (*P =* 0.0006), ZBP1 (*P <*0.0001), and AIM2 (*P =* 0.0004) in the nasopharyngeal epithelial cells from all COVID-19 patients was significantly higher than the healthy control group.

In a similar pattern to that of the nasopharyngeal samples, we found a significant upregulation in expression of MDA5 (*P <*0.0001), ZBP1 (*P =* 0.0007), and AIM2 (*P <*0.0001) transcripts in PBMCs compared with the healthy control group.

We observed a significant difference in the MDA5 and AIM2 transcripts between PBMC and nasopharyngeal samples of all COVID-19 patients; however, there was no significant difference in the ZBP1 transcript between those samples (*P =* 0.08).

### Transcription of target genes in PBMCs and the nasopharyngeal samples of COVID-19 patients based on disease severity.

**Figure 2.**
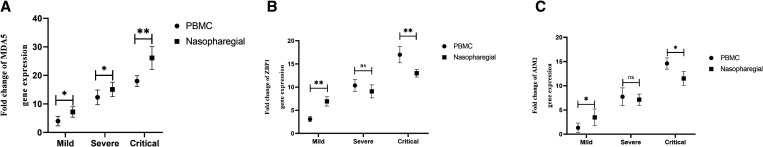
(**A**) Comparison of transcript of MDA5 in both peripheral blood mononuclear cell (PBMC) and nasopharyngeal samples of the COVID-19 patients at different disease intensities. (**B**) Comparison of transcript of ZBP1 in both PBMC and nasopharyngeal samples of the COVID-19 patients at different disease intensity. (**C**) Comparison of AIM2 transcript of in both PBMC and nasopharyngeal sample of the COVID-19 patients at different disease intensities. The data are presented as fold change, and differences in expression between the two groups are indicated by *P <0.05, **P <0.001, and ***P <0.0001. ns = not significant.

There was a significant increase in the MDA5 transcript in nasopharyngeal epithelial cells of COVID-19 patients compared with PBMC samples in the mild disease stage (*P =* 0.01). As the intensity of the disease increased, MDA5 expression increased in nasopharyngeal epithelial cells more than in PBMCs, similar to early stages of the disease. Thus, the level of expression increased in the critical (*P =* 0.002) and severe (*P =* 0.008) disease stages in nasopharyngeal epithelial cells compared with the PBMC samples (Figure 2A).

Moreover, ZBP1 mRNA was significantly elevated in the nasopharyngeal epithelial cells compared with the PBMC samples of COVID-19 patients with mild disease (*P =* 0.0008). We found that as the intensity of the disease increased, the level of ZBP1 mRNA in the PBMC compared with nasopharyngeal epithelial cells increased. Subsequently, the level of its expression in PBMC samples was increased compared with the nasopharyngeal epithelial cells in the critical stage of the disease (*P =* 0.0004) (Figure 2B).

In a pattern similar to that of ZBP1 mRNA expression, the expression of AIM2 in nasopharyngeal epithelial cells was significantly higher than PBMC samples of COVID-19 patients with mild disease (*P =* 0.03). As the intensity of the disease increased, the level of AIM2 mRNA in PBMCs was increased compared with nasopharyngeal epithelial cells. Thus, the level of its expression was increased in the critical stage of the disease in PBMC samples (*P =* 0.009) compared with nasopharyngeal epithelial cells (Figure 2C).

### Transcription of target genes according to disease severity and sex.

**Figure 3.**
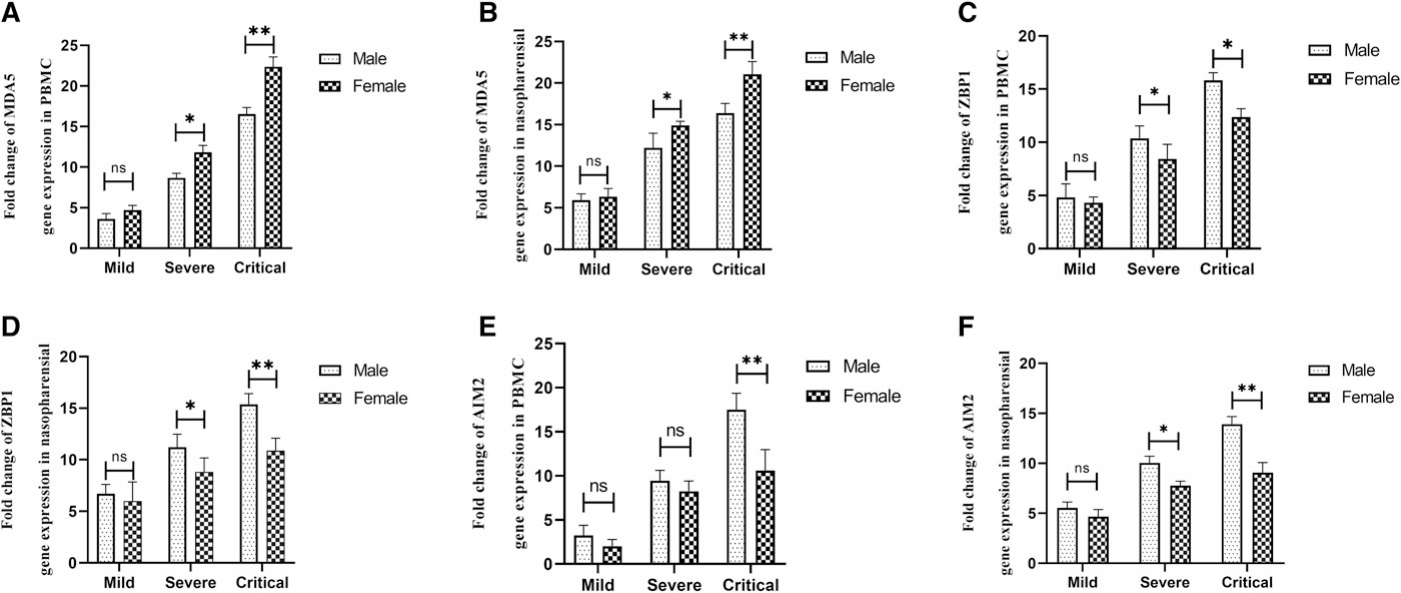
Comparison of ZBP1 transcript in peripheral blood mononuclear cells (PBMC) samples (**A**) and nasopharyngeal (**B**) in males and female at different disease intensities including mild, severe, and critical. Comparison of AMI2 transcript in PBMC samples (**C**) and nasopharyngeal (**D**) in males and female at different disease intensities including mild, severe, and critical. Comparison of MDA5 transcript in PBMC samples (**E**) and nasopharyngeal (**F**) in males and females at different disease intensities including mild, severe, and critical. The data are presented as fold change and differences in expression between the two groups are indicated by *P <0.05, **P <0.001, and ***P <0.0001. ns = not significant.

As disease progressed, we found that female MDA5 transcript was greater than that of male patients. Thus, our results indicated a significant elevated in MDA5 transcript in PBMCs and nasopharyngeal epithelial cells of female patients with critical disease (*P =* 0.0003 and *P =* 0.0009, respectively) and also at the severe stage (*P =* 0.02 and *P =* 0.04, respectively) compared with the male patients (Figure 3A and B).

We found a significant difference in ZBP1 transcript in PBMCs and nasopharyngeal epithelial cells of males at the critical stage (*P =* 0.008 and *P =* 0.0009, respectively) and also at the severe stage (*P =* 0.02 and *P =* 0.009, respectively) of disease compared with female patients (Figure 3B and C).

We also observed AIM2 transcript in PBMCs of males to be significantly higher than those of females at the critical stage (*P =* 0.0002). This was evidenced by a significant upregulation of AIM2 transcripts in nasopharyngeal epithelial cells of male patients with severe (*P =* 0.03) and critical (*P =* 0.0001) disease compared with their female counterparts (Figure 3D and E).

## DISCUSSION

COVID-19 has led to a staggering loss of human life worldwide, resulting in an unprecedented challenge to public health, food systems, and the global workforce.^19^ The process of antiviral responses initiates as acid nucleic viruses is recognized by various PRRs. Although induction of IFNs and anti-inflammatory cytokines against SARS-CoV-2 due to the activation of PRRs can help to clear virus infection, their aberrant production can cause acute respiratory distress syndrome in vulnerable patients.^20^ Several studies have suggested the role of the innate immune sensor in controlling COVID-19 disease, but few studies have examined its expression in respiratory epithelial cells. Our aim in this study was to measure the level of MDA5, ZBP1, and AMI2 mRNA in the nasopharyngeal epithelial cells as well as PBMCs of COVID-19 at different stages of disease to assess the role of these PRR in the antiviral response and the inflammatory settings of COVID-19 patients.

These results indicate that the MDA5 sensor plays a key role in the detection of viral RNA and contributes to the production of type I IFN as an antiviral response. The current study showed a significant difference in MDA5 expression in PBMCs and nasopharyngeal epithelial cells of COVID-19 patients compared with the healthy control group. Moreover, its expression was elevated as the intensity of the disease increased. This research reveals that the production of IFNs due to the PRR activation has an important role in the inhibition of SARS-CoV-2.^21^ There is also evidence on the importance of MDA5 in other coronavirus infections, including Middle East respiratory syndrome coronavirus (MERS-CoV) and severe acute respiratory syndrome coronavirus (SARS-CoV).^22^ Sampaio and colleagues showed that higher production of IFN-I and IFN-III against SARS-CoV-2 is directly due to the MDA5 activation.^23^ Rebendenne and colleagues conducted a study indicating that MDA5 was the key sensor in the SARS-CoV-2 infection in Calu-3 cells as a lung cancer cell line.^24^ Patients with severe COVID-19 may experience chronic overactivation of MDA5 signaling activity, as suggested by another research group,^25^ which confirmed our results. According to these findings, the increase in MDA5 expression in severe or critical COVID-19 patients was probably due to its protective role in interferon production.

The ZBP1 transcript was significantly different between PBMCs and nasopharyngeal epithelial cells of all COVID-19 patients compared with the healthy group. As the intensity of the disease increased, the level of ZBP1 expression likewise increased. As one of the main inflammatory mediators, ZBP1 could recognize DNA and RNA molecules of viruses during their replication or transcription.^11^^,^^26^ Its signaling complex is associated with necroptosis, which induces autonomous activation of genes responsible for producing proinflammatory cytokines.^27^ In this regard, there is evidence that the ZBP1 signaling pathway worsens the condition of COVID-19 patients by damaging lung epithelial cells.^28^ One study proposed that the ZBP1 pathway contributed to the development of cytokine storms in COVID-19 patients by triggering the release of pro-inflammatory cytokines as IP-10 and CXCL-10.^12^ Furthermore, ZBP1 is involved in other viral respiratory diseases such as influenza, MERS-CoV, and SARS-CoV. It has also been reported that ZBP1 could recognize PB1 and NP protein of influenza type A (IAV) and induce inflammatory response and cell death by caspase 8.^29^ Researchers have also found that reduced mortality in ZBP-deficient mice exposed to IAV was due to decreased an inflammatory responses and lung cell damage.^30^ Furthermore, Gutierrez-Alvarez and others discovered that ZBP1 was a crucial gene expressed in both human and mice lung cells after infection with a strain of MERS-CoV and with SARS-CoV.^31^ This finding is in agreement with our results and shows that increased expression of ZBP1 increases the production of pro-inflammatory cytokines, damage to lung epithelial cells, and worsens the clinical condition of patients.

Our findings also showed a significant difference in AIM2 transcript in PBMCs and nasopharyngeal epithelial cells of all patients compared with the healthy group. As the intensity of the disease increased, the AIM2 transcript also increased. AIM2 is crucial in detecting viral DNA, which activates the inflammasome pathway that produces interleukin (IL)-1β and IL-18.^32^ There was a significant elevation in AIM2 molecules in patients of COVID-19. It has also been suggested that the AIM2 molecule is one of the prognostic molecules of SARS-CoV-2 patients for hospitalization.^16^ Colarusso et al showed that AIM2 could trigger inflammation and programmed cell death by processing interleukin IL-1, IL-18, and pro-caspase 1 and activating them.^33^ Other research found a potent correlation between inflammasome-mediated inflammation and Gradermin D in severe COVID-19 cases as opposed to those with milder symptoms. They also proposed that this could be attributed to the high production of AIM2 and highlighted in the immunopathogenesis of COVID-19 patients.^34^ Accordingly, increasing the expression of AIM2 and its signaling might contribute to an uncontrolled inflammatory response, reduce effective antiviral responses, cause tissue damage, and ultimately increase disease severity.

Our study shows that the AIM2 and ZBP1 transcripts were significantly higher than those of PBMCs in the nasopharyngeal epithelial cells of all patients in the early phase of the disease (mild stage). This suggests a possible site-specific and local immune response in the respiratory tract during the early stages of the disease. However, as the disease progressed and became more severe, the ZBP1 and AIM2 transcript in PBMCs increased significantly, illuminating the systemic immune response in the body. Regarding MDA5, along with increasing severity of the disease, its expression level also increased, so that at the critical stage, the level of MDA5 in nasopharyngeal epithelial cells was significantly higher than that of PBMCs. This finding coincides with previous data linking ZBP1 expression to viral infections, specifically influenza A infection.^35^ A previous study indicated that patients with severe disease who were hospitalized in the ICU had signs of hyperactivation of some PRRs, along with increased levels of nucleic acid–sensitive PRR agonists in their blood. Consequently, using nanoparticles to scavenge DAMPs/PAMPs associated with damage may prove advantageous in outcomes in a severe form of COVID-19.^36^ Moreover, there was a correlation observed between the levels of PRR transcript and oxygen saturation levels in patients with COVID-19.^34^ Moreover, research has highlighted the potential for these genes to serve as biomarkers for disease severity in COVID-19 patients.^37^ Also, because SARS-CoV-2 replicates in the cytoplasm of airway epithelial cells, it is essential to consider the role of innate immune responses within airway epithelial cells in COVID-19 infection. These responses are crucial in either eliminating the virus or allowing it to continue infecting the lungs.^38^^,^^39^ These findings are significant because they suggest that the immune response to COVID-19 may be different depending on disease severity. This could have important implications for the development of therapies for COVID-19 because treatments may need to be tailored to the specific stage and severity of the disease.

This study uncovered sex-based discrepancies in clinical outcomes and could underline the possibility of potential targeted therapeutic interventions. Our research shows that male COVID-19 patients exhibit increased expression of AIM2 and ZBP1 genes, which could be a crucial factor in the sex-specific immune reactions in this disease. Interestingly, the study also found a significant increase in ZBP1 and AIM2 transcript among male patients with severe and critical conditions compared with female patients who had similar disease severity. This raises questions about the potential role of gender in COVID-19 disease progression and suggests that these genes may be influenced by hormone levels or other gender-related factors.^40^ A comprehensive analysis has revealed a stark contrast in the likelihood of mortality, ICU admission, and hospitalization between male and female patients. The data conclusively demonstrates that male patients face odds nearly 3 times higher for these adverse outcomes compared with their female counterparts. The underlying mechanisms have not been fully determined; however, factors such as biology, behavior, and environment have been hypothesized to play a role in this disparity.^41^ Research also indicates that females tend to possess higher levels of antibodies, cytokines, and T-lymphocytes compared with males. This discovery offers a plausible explanation for why females typically have a more positive prognosis during viral infections,^42^ and this is consistent with our findings because the expression level of MDA5 as one of the IFN producers to clear the virus is higher in females than in males. Moreover, some research suggests that estrogen may play a role in regulating immune responses because it enhances antiviral immunity.^43^ These findings have the potential to guide the development of gender-specific treatments and aid future studies aimed at understanding the pathogenesis of COVID-19. However, the study has some limitations, such as a small sample size, that may restrict the generalizability of the findings. The research was conducted in a single center, which could lead to selection bias.

## CONCLUSION

The results of this study indicate that there is an elevation in the transcription of MDA5, ZBP1, and AIM2 genes in COVID-19 disease. This suggests that these genes could potentially have significant implications for the severity of COVID-19. The expression level of immune system sensors increases in the early stages of the disease in the epithelial cells of the lung and in the severe stages of the disease in the PBMCs, which may be due to systemic immune response. In addition, increased expression of MDA5 in females may be due to stronger and more appropriate immune responses against SARS-CoV-2, and elevated expression of AIM2 and ZBP1 genes might be associated with the more severe manifestation of COVID-19 in male patients. We recommend that further studies be conducted to clarify this issue.
